# SOuLMuSiC, a novel tool for predicting the impact of mutations on protein solubility

**DOI:** 10.1038/s41598-025-11326-x

**Published:** 2025-07-29

**Authors:** Simone Attanasio, Jean Kwasigroch, Marianne Rooman, Fabrizio Pucci

**Affiliations:** 1 Computational Biology and Bioinformatics, Université Libre de Bruxelles, Ixelles, 1050 Belgium; 2 Interuniversity Institute of Bioinformatics in Brussels, Brussels, 1050 Belgium

**Keywords:** Computational biology and bioinformatics, Computational models, Protein design, Protein folding, Proteome informatics

## Abstract

Protein solubility problems arise in a wide range of applications, from antibody development to enzyme production, and are linked to several major disorders, including cataracts and Alzheimer’s diseases. To assist scientists in designing proteins with improved solubility and better understand solubility-related diseases, we introduce SOuLMuSiC, a computational tool for the fast and accurate prediction of the impact of single-site mutations on protein solubility. Our model is based on a simple artificial neural network that takes as input a series of features, including biophysical properties of wild-type and mutated residues, energetic values computed using various statistical potentials, and mutational scores derived from protein language models. SOuLMuSiC has been trained on a curated dataset of about 700 single-site mutations with known solubility values, collected and manually verified from original literature. It significantly outperforms current state-of-the-art predictors in strict cross validation: the Spearman correlation reaches 0.5 when solubility changes are represented categorically; for the subset with quantitative values, it increases to 0.7. SOuLMuSiC also shows good performance on external datasets containing high-throughput enzyme solubility-related data as well as protein aggregation propensities. In summary, SOuLMuSiC is a valuable tool for identifying mutations that impact protein solubility, and can play a major role in the rational design of proteins with improved solubility and in understanding genetic variants’ effect. It is freely available for academic use at http://babylone.ulb.ac.be/SoulMuSiC/.

## Introduction

Solubility is a key biophysical property of proteins, the lack of which is often a major bottleneck for their production and storage^1–3^. Numerous applications in protein structure determination^1^, pharmaceutics (e.g., production of protein-based therapeutics)^4,5^, and biotechnology (e.g., protein heterologous expression)^6,7^ require high-concentration protein formulation. Moreover, problems related to poor protein solubility and aggregation are central in a wide series of protein misfolding-related diseases, also known as proteinopathies. For example, Alzheimer’s and Parkinson’s diseases^8,9^ are characterized by the growth of insoluble deposits of misfolded proteins, cataracts are related to a decrease in the solubility of human $\gamma$-crystallin^10,11^, and amylin (Islet amyloid polypeptide - IAPP) is involved in diabetes^12^. The different solubility states of these proteins are also important for understanding how they spread and propagate between cells^13^.

Despite the crucial importance of protein solubility and the considerable efforts made by the scientific community, a full comprehension of the mechanisms behind protein solubility remains out of reach. Solubility is influenced by a complex interplay of intrinsic factors such as residue-residue interactions, protein flexibility, amino-acid composition, and hydrophobicity, as well as extrinsic variables such as pH, environmental temperature, ionic strength, and protein concentration^2,14–17^.

In recent decades, multiple experimental and computational approaches have been developed to engineer proteins with improved solubility properties^3,18,19^. For example, when inclusion bodies form in heterologous expression, experimental protocols, including the solubilization of these bodies through the addition of denaturant compounds followed by the refolding of the solubilized proteins, have been designed to get bioactive recombinant proteins^20,21^. Another experimental approach to enhance the solubility of a target protein involves fusing it with solubility-enhancing tags, even though this requires additional chromatographic steps to obtain tag-free recombinant proteins^22^.

All these procedures remain, however, labor-intensive and quite unsatisfactory. Therefore, computational methods have been designed to speed up and complement the experimental approaches. A series of methods for predicting the solubility of a full protein have been developed, which leverage experimental solubility data^23^ to train computational models^15,24–30^.

The challenge of predicting the impact of mutations on protein solubility has been underinvestigated due to the scarcity of mutagenesis data. Indeed, only recently has a database of mutations with known changes in solubility been collected^31^. Moreover, the variability in environmental conditions and in the methods used for variant characterization make constructing datasets and testing variant predictors challenging.

Over the past decade, a few methods have been developed to address this challenge, ranging from simple linear combinations of features to machine learning approaches, either considering the amino-acid sequence or the three-dimensional structure. Renowned methods include CamSol^32^, SODA^33^ and PON-Sol2^34^. However, these methods do not achieve satisfactory performance and moreover, the limited datasets they use for training make them prone to overfitting. A rigorous benchmark of their performances does not exist at the moment in the literature. For these reasons, we proceeded in this study to collect and manually curate a new dataset of mutations with an experimentally determined solubility value, and used it to develop a new method called SOuLMuSiC for the prediction of the impact of mutations on solubility. We compared SOuLMuSiC’s performance with the above mentioned algorithms and tested its ability to generalize to unseen data.

## Methods

### Dataset construction and curation

We collected a set of mutations whose effects on protein solubility have been experimentally characterized. Although protein solubility can be rigorously defined in thermodynamics as the protein concentration in a saturated solution that is in equilibrium with a solid phase^2^, its experimental measurement is far from trivial. Different techniques were used to estimate the solubility of proteins, either directly or indirectly^35,36^. They include measuring protein activity in a pellet after centrifugation^37^, sodium dodecyl sulfate–polyacrylamide gel electrophoresis (SDS-PAGE)^38^, and dynamic light scattering^39^.

To set up our first mutation dataset $\mathcal {D}_{Sol}$, we started to collect mutations and their experimentally measured solubility changes :

1 $$\begin{aligned} \Delta S = S_{\text {mut}} - S_{\text {wt}}, \end{aligned}$$

from the mutational solubility database SoluProtMutDB^31^. We then performed a literature search involving manually reviewing and curation of each SoluProtMutDB entry in the original literature to correct possible errors. Additionally, we extended the search to incorporate recent literature and patent data that were not included in SoluProtMutDB. We excluded data originating from high-throughput techniques such as fluorescence-activated cell sorting, and from mutations in membrane proteins. We focused exclusively on single-site mutations. This led to a total of 702 curated entries in $\mathcal {D}_{Sol}$.

Due to the heterogeneity of experimental approaches and the dependence of solubility on the environmental conditions used in the experiments, such as pH, temperature, and ion buffer, the quantitative values of the changes in solubility upon mutation are often not precisely reported in the original articles. Only 225 entries out of 702 have a numerical estimation of $\Delta S$ (in %). The remaining entries are annotated by their authors with qualitative labels such as ’strongly decrease/increase solubility’, ’decrease/increase solubility,’ or ’no impact.’ We therefore decided to use five discrete solubility scores (−3, −1, 0, 1, 3) or equivalently, five solubility classes (− −, −, =, +, ++). We classified the effects of mutations into these classes according to qualitative labels or, when available, based on their reported $\Delta S$ values, as defined in Table 1. Negative solubility scores represent a decrease in solubility upon mutation and positive values, an increase. A value of zero indicates no significant change in solubility. We also defined the subset of $\mathcal {D}_{Sol}$ that contains the 225 entries with quantitative $\Delta S$ values, called $\mathcal {D}_{Sol}^Q$.

**Table 1:** Correspondence between quantitative solubility changes upon mutations (in %) and qualitative solubility scores and classes.

$\Delta S$	$\Delta S$ scores	$\Delta S$ classes
$\Delta S \le -50$%	−3	− −
−50% $< \Delta S \le$ −10%	−1	−
−10% $< \Delta S<$ +10%	0	=
+10% $\le \Delta S<$ +50%	1	+
$\Delta S \ge -50$%	3	++

We collected the 3-dimensional (3D) structures of all wild-type proteins in $\mathcal {D}_{Sol}$. We considered the experimental structures in the Protein Data Bank (PDB)^40^ when available, but only if their resolution was smaller than 2.5 Å and their sequence 100% identical to the one considered. Otherwise, we modeled the structure using AlphaFold 2^41^. In the few cases in which the oligomeric structures were too large to be modeled with AlphaFold, we used the homology modeling algorithm SWISS-MODEL^42^. The final dataset $\mathcal {D}_{Sol}$ consists of 702 mutations with experimentally characterized effects on protein solubility, inserted in 80 proteins with experimental or modeled structures. For more details about the $\mathcal {D}_{Sol}$ dataset, see Supplementary Section 1.

We set up a second dataset, called $\mathcal {D}_{Inv}$, containing the reverse mutations of $\mathcal {D}_{Sol}$. More precisely, for each mutation (wt $\rightarrow$ mut) in $\mathcal {D}_{Sol}$ with a given $\Delta S$ value, we included the reverse mutation (mut $\rightarrow$ wt) in $\mathcal {D}_{Inv}$ and assigned it a value of $-\Delta S$. We created this dataset to test the antisymmetry properties of our predictor, as non-symmetric models that are biased towards the training set are often constructed, as shown in^43^. The structures of the 702 mutant proteins in $\mathcal {D}_{Inv}$ were modeled with the homology algorithm Modeller^44^ using as template the structures contained in $\mathcal {D}_{Sol}$.

As an additional test mutation dataset, called $\mathcal {D}_{LGK}$, we used solubility data of Levoglucosan kinase (LGK) from *Lipomyces starkeyi*. This enzyme catalyzes the phosphorylation of levoglucosan^45^. Deep mutational scanning was performed on LGK using yeast surface display (YSD)^46^: proteins were fused with an N-terminal domain to localize the protein on the outer cell surface, and with a C-terminal epitope tag, which binds to a fluorescent antibody, enabling the identification of only the variants expressed on the cell surface. Misfolded protein variants are less expressed on the cell surface as the proteasome degrades them to ensure protein quality control. In total, $\mathcal {D}_{LGK}$ consists of 6,246 single-site mutations with known solubility values. Note that these values are only partially related to real solubility as the proteasome can also degrade misfolded soluble proteins. The structure that we used for LGK is homodimeric and has the PDB code 4ZFV.

In the last dataset, referred to as $\mathcal {D}_{A\beta }$, we collected the aggregation propensity of variants of A$\beta$42, a protein known to play a key role in the pathogenesis of Alzheimer’s disease. The variant scores were measured^47^ using a yeast-based selection assay in which A$\beta$ is fused to the essential protein dihydrofolate reductase. Consequently, the aggregation of the variants is inversely related to the yeast growth. In total, we collected 790 variants with known changes in aggregation propensity. These scores are only partially related to solubility, as aggregation phenomena differ from poor solubility precipitation. The structure that we used for A$\beta$42 has the PDB code 1IYT.

The datasets $\mathcal {D}_{Sol}$, $\mathcal {D}_{Inv}$, $\mathcal {D}_{LGK}$ and $\mathcal {D}_{A\beta }$, as well as all structures used in this study, can be retrieved from our GitHub repository at github.com/3BioCompBio/SOuLMuSiC.

### Features

In our SOuLMuSiC model, we used two types of features: structure-based and sequence-based. The former are mainly based on statistical potentials, and the latter, on various amino-acid scales and on protein large language models. Below, we provide a brief description of these features.

$\bullet$ **Statistical potentials**, which are well-known mean force potentials extracted from frequencies of associations between sequence elements, *se*, and structure motifs, *st*, in datasets of protein structures^48,49^. Using this formalism, the folding free energy $\Delta W(st, se)$ of the sequence-structure pair (*st*, *se*) can be computed in terms of the probabilities of *se*, *st* and (*st*, *se*) using the inverse Boltzmann law, as defined in the first equality :

2 $$\begin{aligned} \Delta W(st, se) = -k_B T \log \frac{P(st, se)}{P(st) P(se)} \simeq -k_B T \log \frac{F(st, se)}{F(st) F(se)} \end{aligned}$$

where $k_B$ is the Boltzmann constant and $T$ the absolute temperature taken to be room temperature^49^. As shown in the second approximate equality, the probabilities *P* can be estimated from the frequencies *F* of observation of *se*, *st* and (*st*, *se*) in a high-quality, non-redundant dataset of experimental protein 3D structures.

In our model, we used four types of statistical potentials, differing in the structure and sequence elements considered. Their list and characteristics are given in Table 2. Among these four potentials, two describe local interactions along the polypeptide chain and two are distance potentials that describe tertiary interactions and the likelihood of amino acids being separated by specific spatial distances. Note that more potentials can be defined, but we combined some of them to limit the number of free parameters that we have to optimize and thus to avoid overfitting.

Once these potentials were derived, we used them to compute the change in folding free energy $\Delta \Delta G$ between the wild type (wt) and mutant (mut) proteins. Considering for example the distance potentials $\Delta W_{sds}$ defined in Table 2, the associated free energy change $\Delta \Delta G_{SDS}$ is defined as:

3 $$\begin{aligned} \Delta \Delta G_{SDS} = \Delta G_{SDS} (\text {mut}) - \Delta G_{SDS} (\text {wt}) \end{aligned}$$

where

4 $$\begin{aligned} \Delta G_{SDS} = \sum _{i<j}^N \Delta W_{s_id_{ij}s_j} \end{aligned}$$

with *N* the protein length, $s_{i}$ and $s_{j}$ the sequence types of residues *i* and *j*, and $d_{ij}$ the distance between the geometric side chain centers of residues *i* and *j*. The sum is thus over all residue pairs in the protein. Analogous expressions can be derived for the other potentials. In total, we considered four types of folding free energy terms: $(\Delta \Delta G_{SA} + \Delta \Delta G_{SSA})$, $(\Delta \Delta G_{ST} + \Delta \Delta G_{SST})$, $\Delta \Delta G_{SDS}$, and $\Delta \Delta G_{STD}$. They constitute four of the input features of our model.

For technical details on the construction and implementation of statistical potentials,we refer the reader to^50,51^.

**Table 2:** List of statistical potentials used as features in SOuLMuSiC. “Local” means that the descriptors are close along the sequence and “Distance”, that they are not necessarily close. The sequence descriptor used is the amino-acid type *s*, and the sequence elements, *se*, are single amino acids *s* or amino-acid pairs (*s*, *s*). The structure descriptors are: the solvent accessibility *a* of a residue defined as the ratio of its solvent accessible surface area in a given structure and in an extended Gly-X-Gly tripeptide conformation, computed as in^52^; the backbone torsion angle domain *t* as defined in^53^; the distance *d* between the geometric center of the heavy side chain atoms of two residues as defined in^54^. The structure motifs, *st*, considered are *a*, *t*, *d*, and (*t*, *d*). More details can be found in^50,51^.

Type	Descriptors	Potentials	Expression
Local	*a*, *s*	$\Delta W_{sa} + \Delta W_{ssa}$	$-k_B T \left( \log \frac{F(s,a)}{F(s) F(a)} +\log \frac{F(s,s,a)}{F(s,s) F(a)} \right)$
Local	*t*, *s*	$\Delta W_{st} + \Delta W_{sst}$	$-k_B T \left( \log \frac{F(s,t)}{F(s) F(t)} +\log \frac{F(s,s,t)}{F(s,s) F(t)} \right)$
Distance	*d*, *s*	$\Delta W_{sds}$	$- k_B T \log \frac{F(s,s,d)}{F(s,s) F(d)}$
Distance	*d*, *t*, *s*	$\Delta W_{std}$	$- k_B T \log \frac{F(s,t,d)}{F(s,t) F(t,d)}$

- **Amino-acid scale-based features**. We used four sequence-based features that are essentially amino-acid scales, and a fifth one corresponding to the large language model (LLM) ESM-1v. They are described below; the values for the four former scales can be found in our GitHub repository github.com/3BioCompBio/SOuLMuSiC.
  - $\Delta$Hydro is the difference between the hydrophobicity of the mutant and wild-type residues. The hydrophobicity scale we used is taken from^55^. It shows the best correlation with experimental solubility values, compared to the other tested scales such as the Kyte-Doolittle scale^56^ and the Janin scale^57^.
  - $\Delta$Aro is the difference in aromaticity between the mutant and wild-type residues. The aromaticity is considered equal to one for PHE, TYR, and TRP, and equal to zero for all other amino acids.
  - $\Delta$Iso is related to the isoelectric point Iso of the amino acid under consideration. As the solubility of proteins is minimal near the isoelectric pH^58,59^ and our predictions are for neutral pH, i.e. seven, we defined the feature $\Delta$Iso as the difference of (Iso$-7$)$^2$ for mutant and wild-type proteins:
    5 $$\begin{aligned} \Delta \text {Iso}= ( \text {Iso}_{mut} - 7)^2 - ( \text {Iso}_{wt} - 7)^2 \end{aligned}$$
  - $\Delta \Delta$Apaac is defined from the amphiphilic pseudo amino-acid composition. Apaac describes the hydrophobicity and hydrophilicity distribution patterns along the protein chain and therefore includes sequence-order effects^60^. For a given protein, the Apaac per residue type is its frequency normalized by a factor that includes the correlation effects between hydrophobic or hydrophilic residues along the chain. We downloaded the dataset^15^, in which about 11,000 protein sequences were experimentally identified as soluble or insoluble. For each of these two sets, we calculated the Apaac score per amino-acid type using the protr package^61^ and averaged them over all sequences in each of the two sets. The logarithm of the ratio between Apaac scores of soluble and insoluble sets yields the $\Delta$Apaac index. $\Delta \Delta$Apaac is then computed as the difference between the mutant and wild-type $\Delta$Apaac values.
- **Protein Language models (pLM)**. As last sequence-based feature, we leveraged ESM (ESM-1v), a freely available unsupervised pLM machine learning model that predicts the variant effects from the amino-acid sequence. It is based on a 650M parameter transformer language model with zero-shot inference^62^ trained on UniRef90 2020-03^63^. Including this model in our predictors allows us to leverage the evolutionary information embedded in the complex architecture of the pLM model^64^, without the need of performing a multiple sequence alignment for the target protein, thus preserving speed and scalability.

The correlations between the different features used are examined in Supplementary Section 3.

### Artificial neural network models

Our model’s input are the nine features described in the previous subsection: four folding free energy terms computed from statistical potentials: $(\Delta \Delta G_{SA} + \Delta \Delta G_{SSA})$, $(\Delta \Delta G_{ST} + \Delta \Delta G_{SST})$, $\Delta \Delta G_{SDS}$, and $\Delta \Delta G_{STD}$; four sequence-based features that depend on the similarity of the wild-type and mutant amino acids, i.e. their change in hydrophobicity ($\Delta$Hydro), aromaticity ($\Delta$Aro), isoelectric point ($\Delta$Iso), and amphiphilic pseudo amino-acid composition ($\Delta \Delta$Apaac); ESM that represents the predicted effect of the mutation on protein fitness estimated using the ESM-1v pLM.

We chose to combine this set of features using a simple artificial neural network. We explored more complex architectures but they potentially lead to overfitting due to the limited size of the dataset. This point is discussed in Supplementary Section 2. Our approach is analogous to that of the PoPMuSiC and HoTMuSiC algorithms^50,65^, i.e., we defined the perceptron activation functions to be sigmoid functions of the solvent accessibility *A* of the mutated residues. This involves weighting the input features differently in terms of the per-residue solvent accessibility, as different potentials and features of amino acids are known to contribute differently in the protein core and on the surface^66^.

More precisely, the SOuLMuSiC model reads as:

6 $$\begin{aligned} \Delta S^{\text {SOuL}}= & \beta \left[ \alpha _1(A) \left( \Delta \Delta G_{SA} + \Delta \Delta G_{SSA}\right) + \alpha _2(A) \left( \Delta \Delta G_{ST} + \Delta \Delta G_{SST}\right) +\right. \nonumber \\ & + \left. \alpha _3(A) \Delta \Delta G_{SDS} + \alpha _4(A) \Delta \Delta G_{STD}\right] + \nonumber \\ & +\alpha _5(A) \Delta \text {Hydro} + \alpha _6(A) \Delta \text {Aro} +\alpha _7(A) \Delta \Delta \text {Apaac} +\alpha _8(A) \Delta \text {Iso} +\nonumber \\ & +\alpha _9(A) \text {ESM} \end{aligned}$$

where $\beta$ can be equal to 1 or $-1$, depending on whether the input structure is a globular protein or an insoluble supramolecular homopolymer of proteins, such as amyloid fibrils, and $\alpha _i(A)$ $(i=1,.., 9)$ are sigmoid functions expressed as :

7 $$\begin{aligned} \alpha _i(A) = \left( \frac{1}{1+e^{-a_i A + b_i}} + c_i\right) . \end{aligned}$$

This yields 27 free parameters $(a_i, b_i, c_i)$, which were identified by minimizing the root mean square error (RMSE) between experimental and predicted changes in solubility $\Delta S$ upon mutations in the $\mathcal {D}_{Sol}$ dataset used as training set:

8 $$\begin{aligned} \text {RMSE} = \frac{1}{N} \sum _{k=1}^{N} \left( \Delta S_k^{\text {exp}} - \Delta S_k^{\text {SOuL}}\right) ^2. \end{aligned}$$

where *N* is the number of mutations in $\mathcal {D}_{Sol}$. Minimization is performed using the NMinimize routine implemented in^67^, which employs a standard gradient descent algorithm with default settings and random parameter initialization. Performance is evaluated using a strict leave-one-out cross-validation process, where all mutations in one protein are, in turn, excluded from the training set and then predicted.

## Results

### Prediction performances

**Fig. 1:**
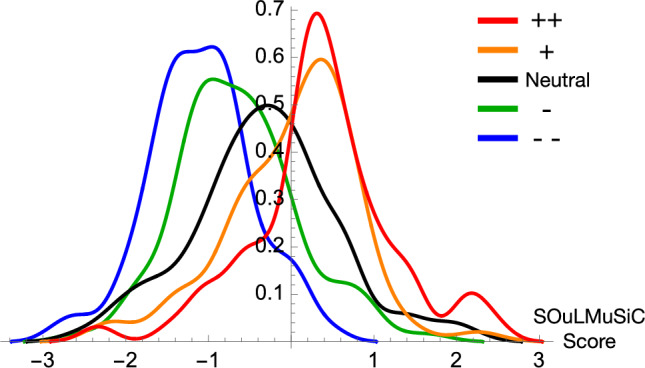
SOuLMuSiC’s score distribution in the $\mathcal {D}_{Sol}$ dataset, obtained in protein-level leave-one-out cross validation, for the five classes of mutations experimentally shown to affect solubility from strongly increasing (++) to strongly decreasing (− −), as defined in Table 1.

We tested the prediction performance of SOuLMuSiC on different datasets. We started with the training set $\mathcal {D}_{Sol}$, and evaluated the performance in leave-one-out cross-validation at protein level. The distributions of the cross-validated SOuLMuSiC scores for all $\mathcal {D}_{Sol}$ entries, separated as a function of their experimentally characterized solubility score defined in Table 1, are shown in Fig. 1. We clearly observe that SOuLMuSiC can distinguish between different solubility classes and identifies with fair accuracy which mutations make proteins more soluble or less soluble.

One of the metrics that we used to evaluate the overall performance of SOuLMuSiC is the Spearman correlation coefficient $\rho$ between the predicted and experimental $\Delta S$ score values ($-3$, $-1$, 0, 1, 3). SOuLMuSiC achieves a $\rho$ value of 0.49 in the dataset $\mathcal {D}_{Sol}$ with the above-mentioned strict cross-validation procedure. This is a fairly strong correlation considering the variability in experimental setups used to determine the $\Delta S$ values.

Notably, when computing the correlation on the subset $\mathcal {D}_{Sol}^Q$ of $\mathcal {D}_{Sol}$ consisting of 225 entries for which a quantitative $\Delta S$ value is available (see Section 2.1), we observe a significantly higher agreement, with a Spearman correlation coefficient of 0.70. This represents a strong performance, especially considering the simplicity of the model and the limited size of the training dataset.

Our SOuLMuSiC model was only trained on single-site mutations of $\mathcal {D}_{Sol}$. Nevertheless, we tested it on a small set of multiple mutations collected from the literature. The correlation coefficient of course dropped but remained good, as shown in Supplementary Section 4.

We also used other performance metrics to evaluate SOuLMuSiC, i.e. multiclass precision, recall, and balanced accuracy (BACC). They are defined and given in Table 3. To convert the SOuLMuSiC score into a multiclass prediction, we set thresholds at the midpoint between the average SOuLMuSiC scores of each pair of adjacent classes. The global value of each metric is obtained by averaging the metric value over each of the five classes.

The global BACC, that in the case of multiclass classification is defined as the average recall, is equal to 0.36, which has to be compared to the expected BACC of 0.2 for a random prediction by a five-class classifier. Although this score is not outstanding, a closer look at the data reveals that the classes of variants with a significant impact on solubility are well predicted, with recall scores equal to 0.64. Other classes are, however, less accurately predicted, which may be partly due to the way we defined these classes. For example, we labeled a change in solubility as “neutral” if it ranges between −10% and +10% compared to the wild type, while the “+” and “−” classes cover changes between +10% to +50% and −10% to −50%, respectively. Given that solubility values can greatly vary according to the experimental methods and environmental conditions, it is unsurprising that the model has more difficulty distinguishing classes with small changes in solubility values, as some misclassifications may arise from inherent experimental uncertainty.

The neutral class has much better precision and much worse recall, meaning that the number of false negatives is much larger than the number of false positives. This is due to the choice of the classification thresholds, chosen to favor the prediction of the extreme classes over the neutral class.

**Table 3:** Cross-validated five-class prediction accuracy of SOuLMuSiC in the $\mathcal {D}_{Sol}$ dataset. The five classes are (++, +, =, −, − −), as defined in Table 1. Precision is defined as (TP/(TP+FP)) where TP, TN, FP, FN are true positives, true negatives, false positive and false negatives, respectively. Recall is defined as (TP/(TP+FN)) and balanced accuracy (BACC) is defined as the average recall.

Classes	Precision	Recall
++	0.24	0.64
+	0.13	0.15
=	0.48	0.17
−	0.29	0.21
− −	0.39	0.64
Average	0.31	0.36

### Comparison with other predictors

We compared SOuLMuSiC’s performance on the $\mathcal {D}_{Sol}$ dataset with state-of-the-art solubility predictors. Note that, as described in the previous subsection, a strict leave-one-out cross validation was performed to provide a reliable assessment of SOuLMuSiC’s performance and thus ensure a fair comparison with other methods. Most of the latter methods use biophysical data at amino-acid level such as hydrophobicity or isoelectric point, and sequence-based information such as residue propensities to adopt secondary structures or residue flexibility in a given sequence window. Only few use structural information (i.e., SOuLMuSiC, CamSol^32^, SOLart^27^). Recent tools, such as SOuLMuSiC, NetSolP^30^, and PON-Sol2^34^, also integrate pLM-based predictions, following recent advances in the pLM field^62^.

Among the tested prediction methods, only 4 (SOuLMuSiC, PON-Sol2^34^, CamSol^32^, SODA^33^) directly predict solubility changes upon mutations, with the former three providing a continuous score and the latter being a binary classifier. Seven others (Protein-Sol^26^, SoDoPE^15^, SoluProt^28^, ccSOLomics^25^, NetSolP^30^, Skade^29^, SOLart^27^) predict protein solubility values *S*; in these cases, the $\Delta S$ values were obtained by subtracting the *S* values of wild-type and mutated proteins. Two predictors, PoPMuSiC and ESM, predict the folding free energy changes upon mutation ($\Delta \Delta G$s) and the fitness changes upon mutation, respectively.

For all these predictors, we computed the Spearman correlation coefficients $\rho$ between the predicted and experimental $\Delta S$ values for all the entries in the $\mathcal {D}_{Sol}$ dataset. We see that SOuLMuSiC largely outperforms the other predictors, achieving a solid Spearman correlation coefficient of 0.49 in leave-one-out cross-validation. Note that PON-Sol2 is trained on a dataset that largely overlaps with $\mathcal {D}_{Sol}$, while all other predictors are essentially blind to it. Although predictors claim higher accuracy in their papers, when these models are tested on new, larger, and highly curated datasets such as $\mathcal {D}_{Sol}$, their performance often drops significantly.

It must be noted that the number of entries in solubility datasets remains limited (approximately 700 for $\mathcal {D}_{Sol}$ and only a few hundreds for other datasets used in the literature to train models), which hinders the development of sufficiently robust methods and limits the possibility of avoiding overfitting. Moreover, most methods do not predict the change in solubility $\Delta S$ but rather the solubility *S* of the input sequence, a slightly different problem that makes predicting changes in solubility more challenging for them. Indeed, almost half of the predictors mentioned above do not show a statistically significant correlation with $\mathcal {D}_{Sol}$ values and are thus not reported in Table 4. The last three methods tested, ESM-1v, SaProt, and PoPMuSiC—predict changes in fitness (the first two) and thermodynamic stability upon mutation (the latter), which are only partially related to solubility. Despite this, their correlation of 0.3 is surprisingly among the highest after SOuLMuSiC’s correlation, indicating a close relationship of their descriptors with solubility.

**Table 4:** Spearman correlation coefficients $\rho$ between experimental $\Delta S$ values and those predicted by different predictors on the $\mathcal {D}_{Sol}$ dataset. SOuLMuSiC’s scores were computed in leave-one-out cross validation on $\mathcal {D}_{Sol}$; PON-Sol2 is trained on a dataset that largely overlaps with $\mathcal {D}_{Sol}$ and its accuracy is possibly overestimated; the other predictors are essentially blind to $\mathcal {D}_{Sol}$. “Res” means features derived from biophysical data at amino-acid level (e.g., hydrophobicity, isoelectric point); “Seq” means sequence-based features (e.g., residue secondary structure propensity, residue flexibility); “Str” means structural features; “Evo” means evolutionary features; “pLM” means protein language model-based predictions. Here we only report the results of the predictors tested that show a statistically significant correlation with experimental data (P-value < 0.01).

Method	Prediction Types	Feature Types	$\rho$
SOuLMuSiC	$\Delta$S	Str, pLM, Res	0.49
PON-Sol2^34^	$\Delta$S	Evo, pLM, Res, Seq	0.29
CamSol^32^	$\Delta$S	Str, Res, Seq	0.25
Protein-Sol^26^	S	Res, Seq	0.16
SoluProt^28^	S	Res, Seq	0.16
SoDoPE^15^	S	Res	0.14
ESM-1v^62^	Fitness	pLM	0.31
SaProt^68^	Fitness	pLM	0.35
PoPMuSiC^50^	$\Delta \Delta$G	Str	0.30

### Test on reverse mutations and antisymmetry properties

To test SOuLMuSiC’s antisymmetry properties, we evaluated it on the dataset of reverse mutations, $\mathcal {D}_{Inv}$. If the change in protein solubility due to a mutation from protein “wt” to protein “mut” is given by Eq. 1, the reverse mutation from protein “mut” to protein “wt” should, by construction, satisfy

9 $$\begin{aligned} \Delta S_{\text {mut}-\text {wt}} = S_{\text {wt}} - S_{\text {mut}} = -(S_{\text {mut}} - S_{\text {wt}}) = -\Delta S_{\text {wt}-\text {mut}}. \end{aligned}$$

This antisymmetry property has already been thoroughly studied and discussed in the field of predicting changes in protein stability and protein-protein binding affinity upon mutations^43,69,70^.

To quantify how much the predictions deviate from perfect antisymmetric behavior, we introduced two measures^43^. The first, $r_{dir,inv}$, is the Pearson correlation coefficient between the predictions on the datasets of direct and reverse mutations, which is equal to $-1$ for a perfectly antisymmetric predictor. The second score, $\langle \delta \rangle$, is defined as:

10 $$\begin{aligned} \langle \delta \rangle = \frac{1}{N} \sum _{i=1}^N ( \Delta S_{\text {wt-mut}}^i + \Delta S_{\text {mut-wt}}^i), \end{aligned}$$

where the sum is over all *N* mutations of the dataset considered. For a perfectly antisymmetric predictor, $\langle \delta \rangle$ is equal to 0.

**Table 5:** Antisymmetry properties of SOuLMuSiC and of the features used for SOuLMuSiC’s construction, measured by $r_{dir,inv}$ and $\langle \delta \rangle$, and performance evaluated by the Spearman correlation coefficients $\rho _{dir}$ and $\rho _{inv}$. Note that the SOuLMuSiC version used here is trained on the whole $\mathcal {D}_{Sol}$ dataset.

	$r_{dir,inv}$	$\langle \delta \rangle$	$\rho _{dir}$	$\rho _{inv}$
SOuLMuSiC	−0.90	−0.37	0.56	0.49
Amino-acid scales				
$\Delta$Hydro	−1.00	0.00	−0.19	−0.19
$\Delta$Aro	−1.00	0.00	−0.19	−0.19
$\Delta$Iso	−1.00	0.00	0.12	0.12
$\Delta \Delta$Apaac	−1.00	0.00	0.20	0.20
Statistical potentials				
$\Delta W_{st} + \Delta W_{sst}$	−0.90	0.04	−0.27	−0.24
$\Delta W_{sa} + \Delta W_{ssa}$	−0.88	0.03	−0.30	−0.28
$\Delta W_{sds}$	−0.84	0.01	−0.33	−0.29
$\Delta W_{std}$	−0.77	−0.03	0.11	0.11
pLM				
ESM-1v	−0.99	−6.4	0.31	0.30

We evaluated SOuLMuSiC and its features on the datasets of direct and reverse mutations $\mathcal {D}_{Sol}$ and $\mathcal {D}_{Inv}$. Table 5 provides the values of the two metrics $\langle \delta \rangle$ and $r_{dir,inv}$, along with $\rho _{dir}$ and $\rho _{inv}$, the Spearman correlation coefficients between predicted and experimental values on these two datasets. SOuLMuSiC shows rather good performance in terms of antisymmetry with $r_{dir,inv}=-0.90$ and $\langle \delta \rangle = -0.37$. Indeed, $r_{dir,inv}$ is rather close to the expected value of $-1$ and $\langle \delta \rangle$ is small compared to the standard deviation of the predicted score distributions, which is in the 0.8-0.9 range for $\mathcal {D}_{Sol}$ and $\mathcal {D}_{Inv}$. The Spearman correlation coefficients $\rho _{dir}$ and $\rho _{inv}$ of SOuLMuSiC predictions for $\mathcal {D}_{Sol}$ and $\mathcal {D}_{Inv}$ are also good, although somewhat better for the direct mutation set than for the reverse mutation set, with values of 0.56 and 0.49, respectively.

To better understand where the deviation from an antisymmetric behavior comes from, we analyzed the properties of each of the nine components of SOuLMuSiC. The features defined from amino-acid scales are perfectly symmetric but correlate poorly with experimental data ($\rho _{dir}$ and $\rho _{inv}$ between 0.1 and 0.2). The statistical potentials are almost symmetric in terms of $\langle \delta \rangle$. They are, however, not totally symmetric in terms of $r_{inv,dir}$, because they incorporate some inaccuracies in the statistical potential derivation and because the mutated structures are modeled rather than experimental. In contrast, the pLM feature has good antisymmetry properties in terms of $r_{inv,dir}$, but not at all in terms of $\langle \delta \rangle$. It is biased toward negative values, which means that neutral mutations are not defined by a score of 0. Both the statistical potentials and pLM correlate significantly better with experimental data than amino-acid scales on both direct and reverse mutations. Indeed, $\rho _{dir}$ and $\rho _{inv}$ are between 0.2 and 0.3 for all statistical potentials except the “std” potential, and are equal to 0.3 for pLM.

### Application to LGK and trade-off between solubility and stability

As an additional test of SOuLMuSiC, we studied the solubility of Levoglucosan kinase (LGK) from *Lipomyces starkeyi*, an enzyme that catalyzes the phosphorylation of levoglucosan^45^. In more detail, we analyzed the interplay between protein solubility and stability, which generally exhibits a trade-off^71^. In general, increasing thermodynamic stability of proteins tends to reduce their aggregation propensities and make them more soluble, but there are examples of proteins with enhanced stability and reduced solubility^72^.

To analyze this trade-off, we applied our SOuLMuSiC predictor, along with PoPMuSiC^50^, the tool we previously developed to predict the impact of mutations on thermodynamic stability. We compared these predictions with deep mutagenesis scanning data where the effects of amino-acid substitutions on a combination of protein solubility and stability were systematically explored using yeast surface display (YSD)^46^. Solubility and stability are challenging to disentangle in high-throughput data, and that is why the YSD experiment considered measures a combination of both.

The results of the comparison are shown in Table 6. We first compared the results of the SOuLMuSiC and PoPMuSiC scores with the experimental YSD scores^46^, and found good Spearman correlation coefficients of $\rho =0.36$ and $\rho =-0.29$, respectively. Note that the solvent accessibility has also a good Spearman correlation of 0.30, which indicates the tendency of mutations in the protein core to have a larger impact than those at the surface. Note that this trend is, as expected, more marked for stability^73^ than for solubility.

Since the experimental YSD score reflects a mixture of the two quantities, stability and solubility, we combined our prediction scores in the following way:

11 $$\begin{aligned} \text {SOuLPoP} = \frac{\text {SOuL}}{\sigma _S} - \frac{\text {PoP}}{\sigma _P} \quad , \end{aligned}$$

where $\text {PoP}$ and $\text {SOuL}$ are the PoPMuSiC $\Delta \Delta G$ value and the SOuLMuSiC score, respectively, and $\sigma _P$ and $\sigma _S$, their respective root mean square deviations on the LGK dataset. Note the minus sign between the two terms. Indeed, variants that increase stability (with a negative $\Delta \Delta G$ in our conventions) are likely to also increase solubility, resulting in a positive SOuL score. As the YSD score arises from a combination of solubility and stability, we observe that the combined SOuLMuSiC and PoPMuSiC predictions perform better than each individually, reaching the correlation $\rho =0.40$.

Note that solubility and stability are mildly correlated: the Spearman correlation coefficient between the SOuL scores and PoPMuSiC’s $\Delta \Delta G$ values is equal to $\rho =-0.31$ on the LGK dataset. We already observed a similar trend on the $\mathcal {D}_{Sol}$ dataset (Table 4).

**Table 6:** Spearman correlation coefficients $\rho$ between the experimental YSD score on the LGK dataset^46^ and SOuLMuSiC’s and PoPMuSiC’s predictions, the solvent accessibility and the combination of SOuLMuSiC and PoPMuSiC (SOuLPoP) according to Eq. 11.

	YSD score
SOuLMuSiC	0.36
PoPMuSiC	−0.29
Solvent accessibility	0.30
SOuLPoP	0.40

It is well known that mutations near the catalytic site are likely to disrupt enzyme activity but are often stabilizing^74^. To study the catalytic site in more detail, we analyzed the prediction scores for all possible mutations of residues located within a distance of less than 6 Å from the center of the catalytic site and compared them with those further away. We found that mutations in the catalytic site have an average $\Delta \Delta G$ value of 0.47 kcal/mol, which is lower than the 1.20 kcal/mol observed for other residues, suggesting that these regions are inherently less impacted in terms of stability^74^. For solubility, however, there is essentially no difference, with the average score for residues close to and far from the catalytic site being equal to about −1.0.

### Application to aggregation-prone proteins

Protein aggregation is a well-studied yet poorly understood phenomenon that leads to a series of pathological conditions^75,76^. A series of proteins end up in misfolded states that can trigger the formation of supramolecular assemblies such as amyloid fibrils. These insoluble assemblies are often observed as deposits in major neurodegenerative diseases: for example, the aggregation of $\alpha$-synuclein is considered as one of the hallmarks of Parkinson’s disease, the formation of amyloid beta (A$\beta$) plaques and neurofibrillary tangles composed of the Tau protein is strongly involved in the pathogenesis of Alzheimer’s disease, and Huntingtin fibrils are the toxic species involved in Huntington’s disease. Although amyloid formation and protein solubility are distinct phenomena, they are interrelated^32,77^. Thus, we evaluated our SOuLMuSiC predictor on its ability to predict the effects of variants on aggregation-prone proteins.

Aggregation-prone proteins are often characterized by flexible and intrinsically disordered regions, making it difficult to obtain high-quality structures of their folded form^78^. However, obtaining the structure of amyloid fibrils is comparatively easier and indeed, hundreds of them can be found in the PDB.

To study protein aggregation propensities, we applied SOuLMuSiC to the A$\beta$−42 protein, using both the amyloid fibril structure and the protein-in-solution structure as input. When the input structure is an insoluble aggregate, we set the parameter $\beta$ in front of the structural terms in our model to $\beta = -1$ (see Eq. 6). In contrast, SOuLMuSiC applied to folded proteins in solution uses $\beta = 1$. This adjustment is thermodynamically intuitive: stabilizing the native globular form of a protein generally leads to increased solubility, while stabilizing an insoluble form, such as a fibril, further decreases the solubility of the assembly.

**Fig. 2:**
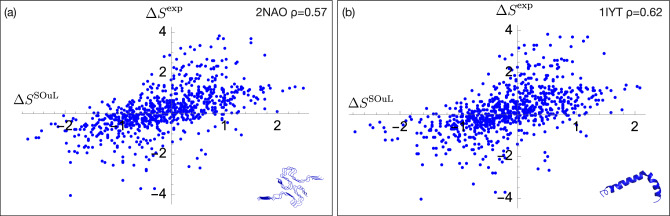
SOuLMuSiC performances on A$\beta$−42 variants using as input (**a**) the fibril structure with PDB code 2NAO and (**b**) the monomeric protein in solution with PDB code 1IYT. The protein structures are shown on the bottom right.

We applied SOuLMuSiC to the structure of the A$\beta$−42 fibrils identified with the PDB code 2NAO^79^ and to the structure of the in-solution A$\beta$−42 protein identified with the code 1IYT^80^. Note that both the 2NAO and 1IYT structures were derived from nuclear magnetic resonance (NMR) data; we used as SOuLMuSiC scores the average of the scores on all 10 NMR structures in the PDB files. We then compared these scores with the solubility data^47^ derived from a yeast aggregation assay combined with deep mutational scanning. We focused on single amino-acid substitutions, excluding truncating and synonymous mutations, which amounts to a total of 790 mutations. We plotted the predicted scores versus the experimental values in Figure 2.

As shown in the figure, the predicted $\Delta S^{SOuL}$ values are well correlated with the experimental $\Delta S^{exp}$ values, with Spearman correlation coefficients of $\rho =0.57$ and 0.62 for the 2NAO and 1IYT structures, respectively. The $\beta$ parameter (in Eq. 6) is set to −1 for the fibril structure 2NAO and to +1 for the solution structure 1IYT. Interestingly, exchanging the $\beta$ values results in a drastic drop in performance, with Spearman correlation $\rho$ decreasing to around 0.2. Also note that the correlation between predictions obtained with the two structures is high with a Pearson correlation coefficient $r=0.84$, although they have completely different conformations.

### Webserver

To make SOuLMuSiC accessible to the broad academic community, we made it available through the webserver http://babylone.3bio.ulb.ac.be/SOuLMuSiC/. The SOuLMuSiC webserver version is the one trained on the entire $\mathcal {D}_{Sol}$ dataset. As SOuLMuSiC is structure-based, users must provide a 3D structure of the target protein as input. The user has the choice among three different methods for this: provide the PDB code^40^ whose structure will be automatically retrieved from the PDB; the UniProt ID, in which case the corresponding structure will be retrieved from AlphaFold DB^81^; or upload his own structure in PDB format. Note that SOuLMuSiC takes into account all chains contained in the submitted structure file when computing the solubility score upon mutations, so users should provide only the chains of interest. The user also has to choose whether the provided structure is a globular protein or a insoluble macromolecular assembly.

Once the structure is submitted, the computation starts. SOuLMuSiC is very fast and performs the prediction of solubility for all single-site mutations in a protein in less than a minute. Upon completion of the calculation, a CSV file containing the results is sent to the email address provided upon submission. The result file contains, for all single-site mutations in all chains of the submitted protein structure, the solvent accessibility of the mutated residue and the $\Delta S^{SOuL}$ value.

## Discussion

The optimization of protein solubility is one of the fundamental goals in any biotechnological process involving proteins. Despite considerable efforts over the last decades, it is still difficult to determine, whether experimentally or computationally, the impact of mutations on protein solubility. In this article, we have taken a step towards this goal by presenting SOuLMuSiC, our tool of the MuSiC suite^50,65,82–84^ dedicated to predict the effect of single-site mutations on protein solubility. Our tool outperforms state-of-the-art approaches when tested on a highly curated dataset of mutations manually collected from the literature and has also been successfully validated on external datasets to assess the generalizability of our approach.

Additional aspects should be explored to further improve our approach. The development of accurate solubility predictors is hindered by the limited availability of mutational data on solubility. Expanding the dataset in the near future could facilitate the training of more complex models with a larger number of features and parameters. Deep mutagenesis data are certainly helpful, though their precise link to protein solubility is complex; solubility, while related to stability and aggregation, is a distinct property, and disentangling these factors is challenging, as we observed in the Results section. Another issue is related to data variability, since solubility measurements are performed using various experimental setups. Even though we made efforts to standardize all collected information in constructing our dataset, this variability remains a source of noise. Finally, the impact of environmental variables, such as pH or temperature, plays a significant role in protein solubility. These factors are not currently taken into account in SOuLMuSiC, and incorporating them could improve its performance.

Although SOuLMuSiC can still be optimized, its current version already achieves good performance and can be used to rationally design new proteins with improved solubility. Given the significant challenges that solubility issues present in both academic and industrial processes, we are confident that SOuLMuSiC will be of great interest to the scientific community and could help optimize a wide variety of biotechnological processes.

## Supplementary Information

Supplementary Information.

## Data Availability

The curated datasets used to train and test our models, as well as all PDB structures analyzed, are available in our GitHub repository: github.com/3BioCompBio/SOuLMuSiC.
