# Supplementary material for: SOuLMuSiC, a novel tool for predicting the impact of mutations on protein solubility
Source: Sci Rep. 2025 Jul 29;15:27531. doi: 10.1038/s41598-025-11326-x (PMC12304109; doi:10.1038/s41598-025-11326-x)
Supplement: Supplementary file 1 — Supplementary Information. [file 41598_2025_11326_MOESM1_ESM.pdf]

Supplementary Material

SOuLMuSiC, a Novel Tool for Predicting the Impact of Mutations on Protein Solubility

Simone Attanasio, Jean Kwasigroch, Marianne Rooman†, Fabrizio Pucci†

[1] *Computational Biology and Bioinformatics, Université Libre de Bruxelles, 1050 Brussels, Belgium*

[2] *Interuniversity Institute of Bioinformatics in Brussels, 1050, Belgium*

June 16, 2025

Contents

1 Dataset statistics **2**

2 Exploring other machine learning architectures **3**

3 More on feature analysis **4**

4 SOuLMuSiC application to multiple-site mutations **5**

1 Dataset statistics

We analyzed in more detail the ΔS distributions in the different datasets considered in this paper. As shown in Table S1, $\mathcal{D}_{Sol}$ is relatively well balanced. The only notable trend is that mutations improving solubility are generally less frequent than those that decrease it, as expected. However, the ratio is not too unfavorable, with the number of mutations improving solubility (++ and + classes) being 2.3 times lower than those destabilizing it (- and - - classes).

Class	# Mutations	Average ΔS in % (#)
- -	145	-81.0 (61)
-	170	-33.2 (47)
=	255	-1.2 (63)
+	71	29.0 (26)
++	61	134.5 (28)
Total	702	-9.1 (225)

Table S1: Statistics in $\mathcal{D}_{Sol}$. Number of mutations and average ΔS value for each solubility class; the average value was calculated on the entries with numerical ΔS values; their number is indicated in parentheses.

For $\mathcal{D}_{Inv}$, the statistics are exactly the same as those shown in Table S1, except that the positive and negative classes are inverted. This is due to the construction of the dataset, where each entry in $\mathcal{D}_{Inv}$ corresponds to the reverse of a mutation present in $\mathcal{D}_{Sol}$.

In Figure S1, we present the distribution of the solubility scores and aggregation propensities for the two datasets $\mathcal{D}_{LGK}$ and $\mathcal{D}_{A\beta}$. The distribution of the former is bimodal and clearly unbalanced, with a substantial number of the 6,246 mutations either decreasing solubility or neutral, and very few mutations improving solubility. In contrast, the distribution for $\mathcal{D}_{A\beta}$ (790) is more symmetric, with a similar number of mutations increasing (440 mutations) or decreasing (350 mutations) the aggregation propensity. Note that these datasets, unlike $\mathcal{D}_{Sol}$ and $\mathcal{D}_{Inv}$, result from high-throughput experiments involving systematic saturation mutagenesis, with almost all possible single-site mutations tested.

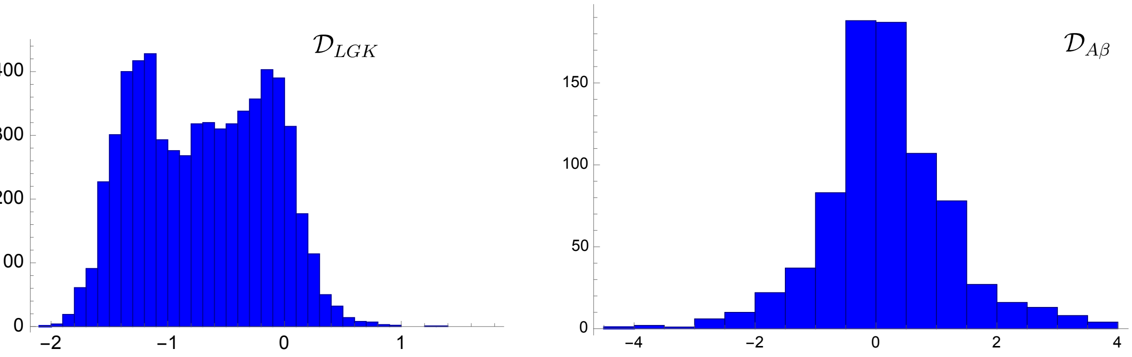

Figure S1: Distribution of the solubility score and aggregation propensity for the datasets $\mathcal{D}LGK$ and $\mathcal{D}A\beta$, respectively.

2 Exploring other machine learning architectures

We compared the artificial neural network architecture of SOuLMuSiC, shared by other tools in the MuSiC suite (PoPMuSiC [1], HoTMuSiC [2], SNPMuSiC [3]), with other machine learning architectures. SOuLMuSiC model's simplicity, characterized by the absence of hidden layers and the use of a sigmoid activation function modulated by solvent accessibility, contributes to its robustness and interpretability. This choice of design not only reduces the risk of overfitting, especially given the limited size of available training data, but also aligns with the biophysical rationale underlying the predictors.

We show in Table S2 that, despite its simplicity, SOuLMuSiC's artificial neural network model achieves competitive performance, outperforming other non–physics-informed networks in terms of Pearson correlation when evaluated in cross validation.

Model	r DirectV	r CrossV
SOuLMuSiC	0.56	**0.49**
Standard Feed-Forward Neural Network	0.67	0.36
Random Forest	**0.76**	0.42
GradientBoostedTrees	0.64	0.39
K-Nearest Neighbors	0.56	0.43

Table S2: Effect of various model architectures on the Pearson correlation coefficient r between predictions and experimental values from $\mathcal{D}_{\text{Sol}}$ in both direct and cross validation.

3 More on feature analysis

We analyzed the Pearson correlations between the different features included in the SOuLMuSiC model to assess potential redundancy. In Figure S2, we present the pairwise correlation matrix computed for the nine input features. We observe that all pairwise correlations remain below 0.40 (in absolute value), except those of three pairs of sequence-based features: ΔHydro-ΔIso, ΔHydro-ΔAro, and ΔHydro-ΔΔApaac, which show moderate correlations of 0.66, -0.47, and -0.66, respectively. It must be emphasized that the generally low correlations, especially between structure-based and sequence-based features, indicate that the two types of features capture largely complementary information. This supports the rationale for including both sequence- and structure-based inputs in the model.

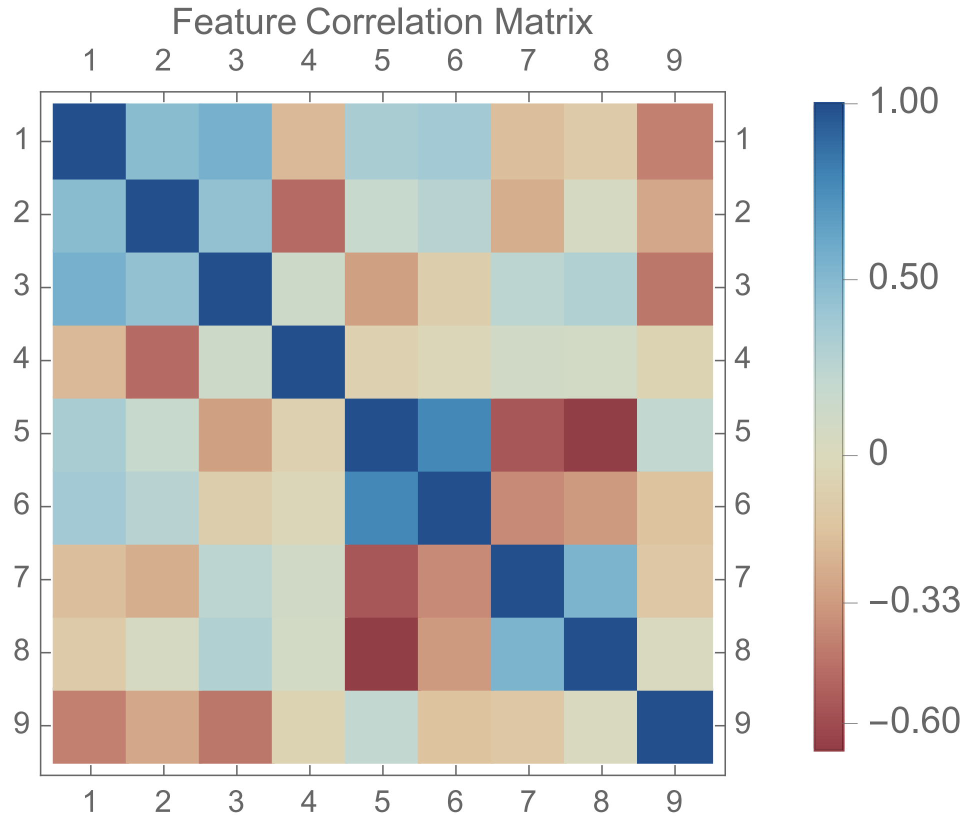

Figure S2: Pearson correlation matrix between the different features of rthe model : 1. $(\Delta\Delta G_{SA}+\Delta\Delta G_{SSA})$, 2. $(\Delta\Delta G_{ST}+\Delta\Delta G_{SST})$, 3. $\Delta\Delta G_{SDS}$, 4. $\Delta\Delta G_{STD}$, 5.ΔHydro, 6. ΔAro, 7.ΔIso, 8. ΔΔApaac and 9. ESM

4 SOuLMuSiC application to multiple-site mutations

Although SOuLMuSiC was originally designed and trained to predict the effects of single-site mutations, we explored how it performs on multiple mutations. To this end, we performed a literature screening to collect data on multiple mutations that were not included in the training set. We compiled a dataset of 36 double and triple mutations with experimentally measured impacts on solubility (this dataset, along with SOuLMuSiC predictions, is available in our GitHub repository). Note that we did not include higher-order mutations (i.e., mutations involving four or more sites) in this analysis.

We then predicted the effect of multiple mutations by simply summing the SOuLMuSiC scores for the individual single mutations, and compared these predictions with the experimentally observed changes in solubility (ΔS). The Spearman correlation between predicted and experimental ΔS values was 0.37 (p-value = 0.02). While this correlation is, as expected, lower than that observed for single-site mutations, it is nonetheless encouraging, especially considering the approximations involved in modeling the effect of multiple mutations as the sum of individual effects.

Although additional terms accounting for epistatic interactions between mutations could be incorporated into the model to further improve predictions on multiple mutations, we leave this extension for a future investigation.

References

[1] Yves Dehouck, Jean Marc Kwasigroch, Dimitri Gilis, and Marianne Rooman. Popmusic 2.1: a web server for the estimation of protein stability changes upon mutation and sequence optimality. *BMC bioinformatics*, 12:1–12, 2011.

[2] Fabrizio Pucci, Raphaël Bourgeas, and Marianne Rooman. Predicting protein thermal stability changes upon point mutations using statistical potentials: Introducing hotmusic. *Scientific reports*, 6(1):23257, 2016.

[3] François Ancien, Fabrizio Pucci, Maxime Godfroid, and Marianne Rooman. Prediction and interpretation of deleterious coding variants in terms of protein structural stability. *Scientific reports*, 8(1):4480, 2018.
